# Supplementary material for: Evaluation of Bacterial Population Changes and Ecological Dynamics in Oil-Impacted Soils Using 16S rRNA Amplicon Sequencing
Source: Biology (Basel). 2025 Aug 18;14(8):1074. doi: 10.3390/biology14081074 (PMC12383895; doi:10.3390/biology14081074)
Supplement: Supplementary file 1 [file biology-14-01074-s001.zip › biology-3791652-supplementary.pdf]

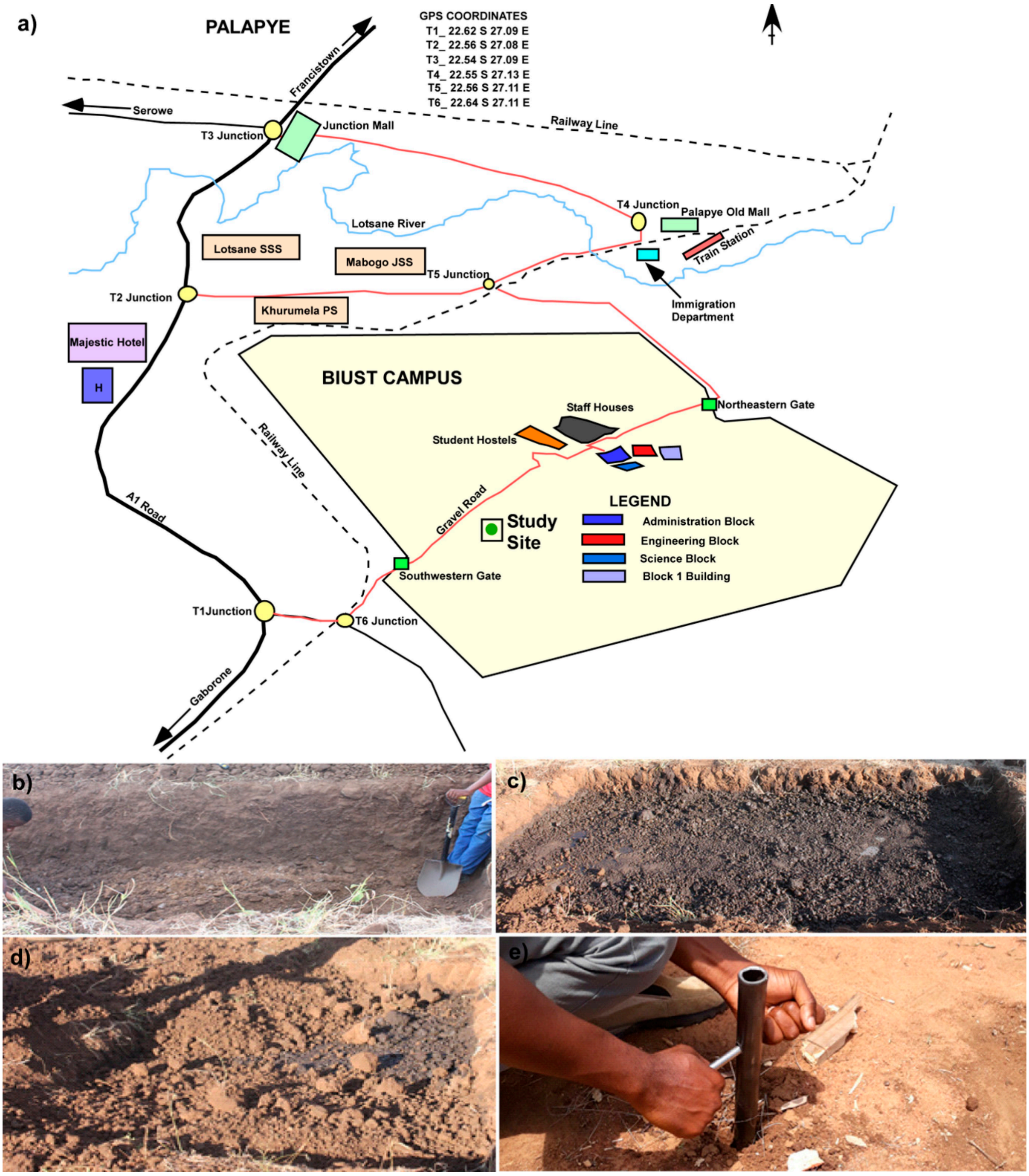

Figure S1. (a) A map showing the location of the study area, (b) excavation of the pit of dimensions 2 m width by 4 m length and 2 m depth, (c) the refill of the pit with the same excavated material and used motor oil spillage surface at a depth of 0.3 m from the ground surface, (d) burial of the used motor oil with the initial excavated material of the area up to the ground level and, (e) photograph of a standard auger planted into the uncontaminated zone of the study area.

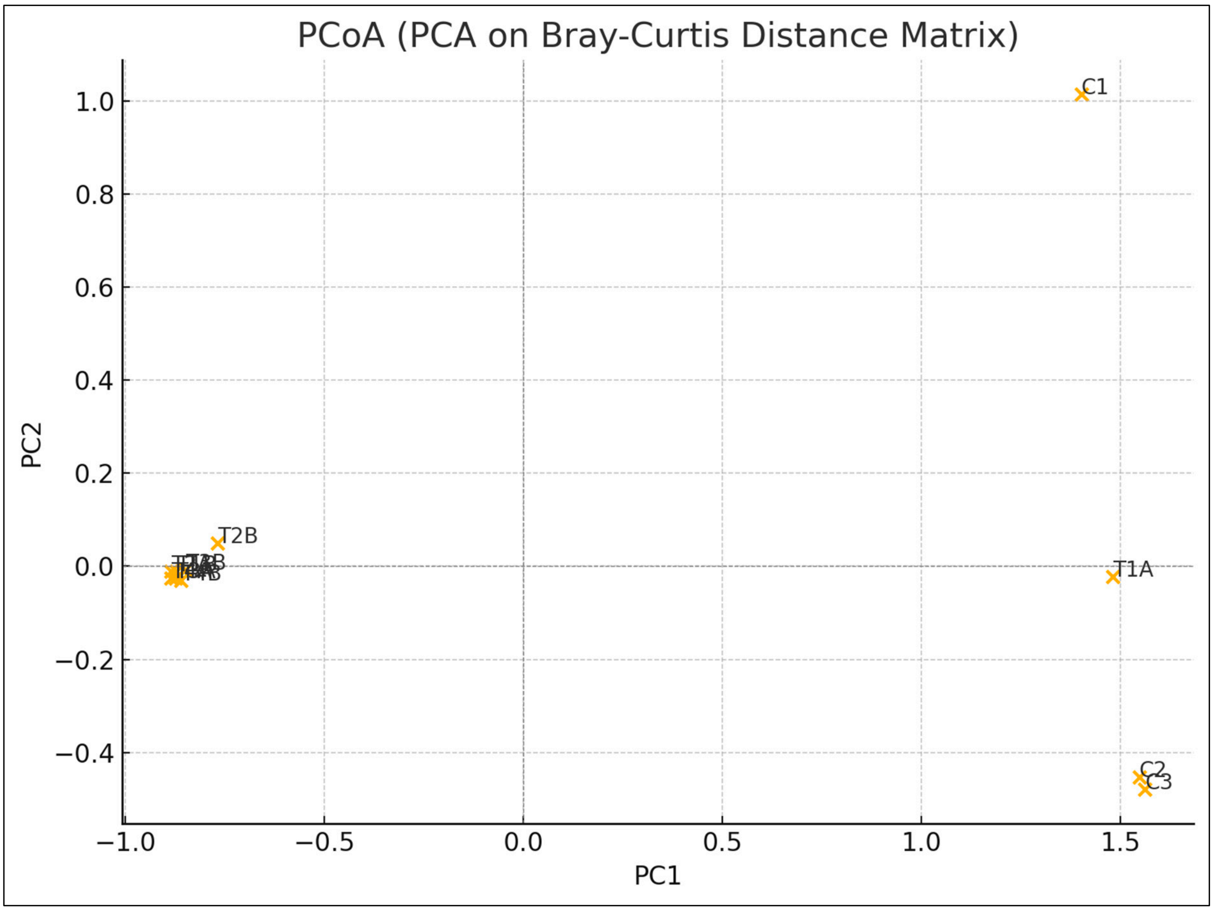

Figure S2. Principal Coordinates Analysis (PCoA) based on Bray–Curtis dissimilarity of microbial communities. Each point represents a sample, with spatial proximity indicating similarity in community composition. The first two coordinates capture the greatest proportion of variation in the dataset.
